# ﻿Morphology and phylogeny reveal two new species of pestalotioid fungi associated with hawthorn in Northeast China

**DOI:** 10.3897/mycokeys.122.153767

**Published:** 2025-09-11

**Authors:** Rong Xu, Wenxin Su, Shangqing Tian, Chitrabhanu S. Bhunjun, Yu Li, Chayanard Phukhamsakda

**Affiliations:** 1 School of Life Science, Jinggangshan University, Ji’an 343009, China; 2 Internationally Cooperative Research Center of China for New Germplasm Breeding of Edible Mushroom, Jilin Agricultural University, Changchun 130118, China; 3 School of Food Science and Engineering, Yangzhou University, Yangzhou 225127, China; 4 School of Science, Mae Fah Luang University, Chiang Rai 57100, Thailand; 5 Center of Excellence in Fungal Research, Mae Fah Luang University, Chiang Rai 57100, Thailand

**Keywords:** *Crataegus pinnatifida*, new species, pathogenicity, saprobes, *
Sporocadus
*, taxonomy

## Abstract

Hawthorn (*Crataegus pinnatifida*), a valuable fruit tree with significant economic and ecological importance, is cultivated across numerous regions in China. In this study, we describe two novel saprobic fungi *Sporocadus changchunensis* and *S. crataegicola*. The novelty of these species is supported by both morphological characteristics and analyses using maximum likelihood (ML) and Bayesian inference (BI). *Sporocadus crataegicola* formed a distinct clade from *S. changchunensis* and *S. rotundatus* with 98% ML and 1.00 BPP statistical support, and the morphological character of *S. crataegicola* is remarkable in its distinguishable shape of ascospores and fewer septa. *Sporocadus changchunensis* differs from *S. italicus*, which was also found on *Crataegus*, by its narrower ostiolar canal, wider hamathecium, and smaller asci. The internal transcribed spacer (ITS) region, the large subunit (LSU) of ribosomal DNA, the RNA polymerase II subunit (*rpb*2), the translation elongation factor 1-alpha (*tef*1-α), and β-tubulin (*tub*2) genes were used for the phylogenetic analyses. An inoculation experiment was also performed to determine the possibility of latent pathogens on *C. pinnatifida*. The inoculation experiment showed that the species caused black spots on the mature leaves of hawthorn. We hypothesize that saprobes possess potential pathogenic ability that may lead to the occurrence of plant diseases under various circumstances. This study also extends the knowledge of the host range and geographic distribution of *Sporocadus* species in China.

## ﻿Introduction

*Crataegus pinnatifida*, commonly known as Chinese hawberry, hawthorn, or Shanzha, is native to Eastern Asia (Edwards et al. 2012). The genus *Crataegus* (Rosaceae, Rosales) comprises approximately 280 species, predominantly found in the northern temperate zones of East Asia, Europe, and eastern North America (Nazhand et al. 2020; Ma et al. 2024). Hawthorn is a widely cultivated, perennial woody plant valued for its red berries and ornamental blossoms (Qin et al. 2022). In China, both the fruit and leaves of *C. pinnatifida* have been extensively used in traditional Chinese medicine for the treatment of cardiovascular diseases and digestive disorders (Wu et al. 2020; Li et al. 2023a). The historical use of *C. pinnatifida* as a herbal medicine dates back to the Tang Dynasty (Su 1981).

Microfungi associated with *Crataegus* have been illustrated in several studies (Zhao et al. 2013; Salazar-Cerezo et al. 2020; Boeraeve et al. 2021; Khalilabad and PhotoHifra 2021). Notably, many fungi such as *Aureobasidium pullulans*, *Botrytis cinerea*, *Monilinia johnsonii*, *Peltaster fructicola*, *Pseudoveronaea ellipsoidea*, *Ramichloridium punctatum*, *Schizothyrium wisconsinense*, *Scleroramularia henaniensis*, and *Trichomerium dioscoreae* have been identified as pathogens affecting *C. pinnatifida* in China, significantly reducing fruit yield (Jing et al. 1982; Chen 2016; Zhang et al. 2018). Therefore, comprehensive surveys are urgently required to identify pathogenic or latent fungi associated with *C. pinnatifida*.

Sporocadaceae is a well-known family that contains pestalotioid taxa in the order Amphisphaeriales. They are characterized by having multi-septate conidia and bearing appendages at one or both end-cells (Jaklitsch et al. 2016; Phukhamsakda et al. 2020; Tian et al. 2022; Hyde et al. 2023). This family was recently revised to include coelomycetous fungi, resulting in the recognition of 30 monophyletic genera (Liu et al. 2019). Razaghi et al. (2024) further expanded this work through a comprehensive reappraisal of the family’s diversity and host associations in China. Members of Sporocadaceae contain many important plant pathogens causing diseases in a wide range of plants worldwide (Maharachchikumbura et al. 2014; Liu et al. 2019; Norphanphoun et al. 2019; Bhunjun et al. 2024a).

*Sporocadus* was erected by Corda (1839) without a designated type species. Hughes (1958) later lectotypified the genus with *S. lichenicola*. Although it was once synonymized with *Seimatosporium* under a broad generic concept (Sutton 1975), *Sporocadus* was later resurrected based on distinct conidial morphology and multi-locus phylogenetic analyses (Brockmann 1976; Nag Raj 1993; Liu et al. 2019). It is the type genus of Sporocadaceae and is primarily characterized by having septate, fusoid, cylindrical, or obovoid conidia, and obovoid conidia typically lacking appendages (except in *S. trimorphus* and *S. rosarum*) (Liu et al. 2019). *Sporocadus* species are commonly saprobes or plant pathogens (Serdani et al. 2010; Bundhun et al. 2021; Liu et al. 2019; Kanetis et al. 2022; Moghadam et al. 2022; Travadon et al. 2022; Razaghi et al. 2024). Currently, there are 26 epithets of *Sporocadus* listed in Species Fungorum (2025).

In this study, we introduce two new species, *Sporocadus changchunensis* and *S. crataegicola*, from dead wood and senescent leaves of *Crataegus pinnatifida* collected in China, with complete descriptions and illustrations. The species were compared morphologically with other *Sporocadus* species. Phylogenetic analyses were conducted to confirm the taxonomic position using maximum likelihood and Bayesian inference of combined ITS, LSU, *rpb*2, *tef*1-α, and *tub*2 sequence data.

## ﻿Materials and methods

### ﻿Sample collection, isolation, and morphology

Dead branches and leaves of *Crataegus pinnatifida* exhibiting conspicuous fungal fruiting bodies were carefully collected in June 2022 from the Jilin Province of China (43°10'N, 124°20'E). This region is located in the Northeast Plain and experiences an annual rainfall of approximately 400–600 mm, characterized by a temperate continental monsoon climate and a mix of coniferous and broad-leaved forests. The samples were placed in plastic bags labeled with the collection details (Rathnayaka et al. 2025) and then transferred to the laboratory. The samples were observed using a Zeiss Stemi 2000C stereomicroscope (ZEISS, Germany) equipped with a Leica DFC450C digital camera (Leica, Germany). Conidial masses were mounted on a slide with a drop of distilled water. Morphological characteristics were observed and photographed using a Zeiss AX10 light microscope fitted with an Axiocam 506 digital camera. Microscopic measurements were performed using the ZEN 3.4 (blue edition) software (ZEISS, Germany). Adobe Photoshop CC2020 (Adobe Systems, USA) was used to process the images.

Pure fungal colonies were obtained using single spore isolation (Senanayake et al. 2020). Germinating spores were transferred aseptically to potato dextrose agar (PDA) and grown at 25 °C. Specimens and pure cultures were deposited in the Herbarium of Mycology, Jilin Agricultural University (**HMJAU**), Changchun, China, and the Engineering Research Center of Edible and Medicinal Fungi, Ministry of Education culture collection (**EMFCC**), respectively. The new taxa were registered in MycoBank (Crous et al. 2004).

### ﻿DNA extraction, PCR amplification, and sequencing

The mycelium was harvested from 2-week-old pure cultures in PDA and frozen using liquid nitrogen. Genomic DNA extraction was performed using a NuClean PlantGen DNA Kit (CWBIO, China) according to the manufacturer’s protocol. The internal transcribed spacer (ITS) region, the large subunit (LSU), the RNA polymerase II second-largest subunit (*rpb*2), the translation elongation factor 1-alpha (*tef*1-α) and beta tubulin (*tub*2) genes were amplified by polymerase chain reaction (PCR) using ITS5/ITS4 (White et al. 1990), LR0R/LR5 (Vilgalys and Hester 1990), fRPB2-5f/fRPB2-7cr (Liu et al. 1999), EF1-728F/EF1-986R (Carbone and Kohn 1999) and T1/Bt2b (O’Donnell et al. 1997) primers. The amplification reactions were performed using 20 μl PCR mixtures containing 9 μl sterilized water, 10 μl of 2×Es Taq MasterMix (Dye), 0.3 μl (10 µM) of forward and reverse primers, and 0.4 μl (200 ng/µL) of DNA template. The amplification conditions for ITS, LSU, and *tub*2 were as follows: 94 °C for 5 min, followed by 35 cycles of denaturation at 94 °C for 30 sec, annealing at 53 °C for 45 sec, and elongation at 72 °C for 90 sec, with a final extension at 72 °C for 10 min. The amplification conditions for the *rpb*2 and *tef*1-α were as follows: initial denaturation at 95 °C for 5 min, followed by 35 cycles at 95 °C for 30 sec, annealing at 56 °C for 45 sec, and elongation at 72 °C for 90 sec, with a final extension period of 72 °C for 10 min. The PCR products were confirmed by electrophoresis on 1% agarose gels stained with a standard DNA dye. Purification and sequencing of amplified PCR fragments were carried out by Sangon Biotech Co, Shanghai, China.

### ﻿Sequencing and sequence alignment

Sequences obtained from this study were subjected to BLASTn searches in GenBank (http://blast.ncbi.nlm.nih.gov/), and the newly generated nucleotide sequences were deposited in GenBank. Reference sequence data were downloaded following recent publications as detailed in Table 1 (Kanetis et al. 2022; Peng et al. 2022). The sequences were aligned using MAFFT v. 7 and ambiguous nucleotides were manually adjusted in AliView where necessary (Katoh and Standley 2013; Larsson 2014). The sequence datasets were combined using SequenceMatrix (Vaidya et al. 2011).

**Table 1.** Taxa used in the phylogenetic analyses and their corresponding GenBank accession numbers. The ex-type strains are indicated in bold, and the newly generated sequences are in blue. “-” represents data that is not available.

Species	Culture collection no.	Host	Country	ITS	LSU	*rpb*2	*tef*1-α	*tub*2	Reference
***Allelochaeta biseptata***	**CBS 131116**	***Eucalyptus oresbia***	**Australia**	**MH554075**	**MH554286**	-	**MH554510**	**MH554749**	(Liu et al. 2019)
***Allelochaeta fusispora***	**CBS 144172**	***Eucalyptus* sp.**	**Australia**	**MH554094**	**MH554304**	-	**MH554528**	**MH554767**	
***Distononappendiculata banksiae***	**CBS 143906**	***Banksia formosa***	**Australia**	**MH554158**	**MH554354**	**MH555057**	**MH554593**	**MH554831**	
***Pseudopestalotiopsis indica***	**CBS 459.78**	***Hibiscus rosa-sinensis***	**India**	**KM199381**	**MH554263**	**MH554963**	**KM199560**	**KM199470**	
***Pseudopestalotiopsis thailandica***	**MFLUCC 17-1724**	***Rhizophora mucronata***	**Thailand**	**MK764292**	**MK764314**	-	**MK764336**	**MK764358**	(Norphanphoun et al. 2019)
***Seimatosporium botan***	**H4619**	***Paeonia suffruticosa***	**Japan**	**AB594799**	**AB593731**	-	-	**LC047770**	(Hatakeyama and Harada 2004)
***Seimatosporium botan***	**JCM 12837**	-	-	**LC228665**	**LC228723**	-	-	-	
***Seimatosporium chinense***	**CFCC 70988**	***Rosa xanthina***	**China**	**PQ279535**	**PQ279531**	**PQ283812**	**PQ287323**	**PQ287325**	(Yang et al. 2024)
*Seimatosporium chinense*	N001B	*Rosa xanthina*	China	PQ279536	PQ279532	PQ283813	PQ287324	PQ287326	
***Seimatosporium cyprium***	**L111**	***Vitis vinifera***	**Cyprus**	**ON680684**	**ON705769**	-	**ON863790**	**ON695856**	(Kanetis et al. 2022)
*Seimatosporium cyprium*	L112	***Vitis vinifera***	Cyprus	ON695889	ON692404	-	ON863791	ON695848	
*Seimatosporium discosioides*	H4621	*Punica granatum*	Japan	AB594800	AB593732	-	-	-	(Lawrence et al. 2018)
***Seimatosporium endophyticum***	**GUCC 194105.1**	***Rosa roxburghii***	**China**	**ON791165**	**ON791208**	-	**ON815938**	**ON815975**	(Zhang et al. 2023)
*Seimatosporium endophyticum*	GUCC 194105.2	*Rosa roxburghii*	China	ON791166	ON791209	-	ON815939	ON815976	
***Seimatosporium germanicum***	**CBS 437.87**	-	**Germany**	**MH554047**	**MH554259**	**MH554957**	**MH554482**	**MH554723**	(Liu et al. 2019)
*Seimatosporium luteosporum*	Napa754	*Vitis vinifera*	USA	KY706283	KY706308	-	KY706333	KY706258	(Lawrence et al. 2018)
***Seimatosporium luteosporum***	**CBS 142599**	***Vitis vinifera***	**USA**	**KY706284**	**KY706309**	-	**KY706334**	**KY706259**	
*Seimatosporium marivanicum*	IRAN 2300C	*Vitis vinifera*	Iran	MW361951	MW361959	-	MW375357	MW375351	(Moghadam et al. 2022)
*Seimatosporium parasiticum*	NBRC 32682	*Physocarpus amurensis*	Japan	AB594808	AB593741	-	-	-	(Nag Raj 1993)
***Seimatosporium physocarpi***	**MFLUCC 14-0625**	***Physocarpus opulifolius***	**Russia**	**KT198722**	**KT198723**	**MH554917**	**MH554434**	**MH554676**	(Norphanphoun et al. 2015)
***Seimatosporium pistaciae***	**CBS 138865**	***Pistacia vera***	**Iran**	**KP004463**	**KP004491**	**MH554915**	**MH554432**	**MH554674**	(Liu et al. 2019)
***Seimatosporium rosae***	**MFLUCC 14-0621**	***Rosa canina***	**Czechoslovakia**	**KT198726**	**KT198727**	**LT853153**	**LT853203**	**LT853253**	
***Seimatosporium soli***	**CBS 941.69**	**Soil**	**Denmark**	**MH554071**	**MH554282**	**MH554983**	**MH554507**	-	
***Seimatosporium tibetense***	**CGMCC 3.23503**	-	**China**	**OR247936**	**OR247954**	**OR380975**	**OR361511**	**OR381084**	(Razaghi et al. 2024)
*Seimatosporium tibetense*	LC15857	-	China	OR247937	OR247955	OR380976	OR361512	OR381085	
*Seimatosporium tostum*	NBRC 32626	-	-	AB594795	AB593727	-	-	-	(Tanaka et al. 2011)
*Seimatosporium vitifusiforme*	Wint753	*Vitis vinifera*	USA	KY706296	KY706321	-	KY706346	KY706271	(Lawrence et al. 2018)
*Seimatosporium vitifusiforme*	Napa768	*Vitis vinifera*	USA	KY706285	KY706310	-	KY706335	KY706260	
***Seimatosporium vitis***	**MFLUCC 14-0051**	***Vitis vinifera***	**Italy**	**KR920363**	**KR920362**	-	-	-	(Senanayake et al. 2015)
*Seimatosporium vitis*	Napa764	*Vitis vinifera*	USA	KY706273	KY706298	-	KY706323	KY706248	
*Seimatosporium vitis*	Wint752	*Vitis vinifera*	USA	KY706279	KY706304	-	KY706329	KY706254	
***Seimatosporium vitis*-*viniferae***	**CBS 123004**	***Vitis vinifera***	**Spain**	**MH553992**	**MH554211**	**MH554901**	**MH554418**	**MH554660**	(Liu et al. 2019)
***Sporocadus biseptatus***	**CBS 110324**	-	-	**MH553956**	**MH554179**	**MH554853**	**MH554374**	**MH554615**	
***Sporocadus brevis***	**CFCC 55170**	***Rosa spinosissima***	**China**	**OK655780**	**OK560371**	**OL742155**	**OL814537**	**OM401659**	(Peng et al. 2022)
*Sporocadus brevis*	ROC 092	*Rosa spinosissima*	China	OK655781	OK560372	OL742156	OL814538	OM401660	
***Sporocadus cavernicola***	**CGMCC 3.23173**	**Soil**	**China**	**OR357758**	**OR247948**	**OR380981**	**OR361499**	**OR381096**	(Razaghi et al. 2024)
*Sporocadus cavernicola*	LC15863	Soil	China	OR357759	OR247949	OR380982	OR361500	OR381097	
***Sporocadus changchunensis***	**EMFCC 0014**	***Crataegus pinnatifida***	**China**	**OR791294**	**OR791292**	**PQ096954**	**PQ096031**	**PQ122556**	This study
***Sporocadus corni***	**MFLUCC 14-0467**	***Cornus* sp.**	**Italy**	**KT162918**	**KR559739**	-	-	-	(Senanayake et al. 2015)
*Sporocadus corni*	MFLUCC 14-1208	*Cornus sanguinea*	Italy	KT868532	KT868531	KU519576	KU519577	-	
*Sporocadus cornicola*	CBS 143889	*Cornus sanguinea*	Germany	MH554121	MH554326	MH555029	MH554555	MH554794	(Liu et al. 2019)
***Sporocadus cotini***	**CBS 139966**	***Cotinus coggygria***	**Russia**	**MH554003**	**MH554222**	**MH554916**	**MH554433**	**MH554675**	
***Sporocadus crataegicola***	**EMFCC 0015**	***Crataegus pinnatifida***	**China**	**OR791295**	**OR791293**	**PQ096955**	**PQ096032**	-	This study
***Sporocadus hyperici***	**CGMCC 3.23174**	***Hypericum* sp.**	**China**	**OR357754**	**OR247944**	**OR380977**	**OR361501**	**OR381092**	(Razaghi et al. 2024)
*Sporocadus hyperici*	LC15844	*Hypericum* sp.	China	OR357755	OR247945	OR380978	OR361502	OR381093	
***Sporocadus incanus***	**CBS 123003**	***Prunus dulcis***	**Spain**	**MH553991**	**MH554210**	**MH554900**	**MH554417**	**MH554659**	(Liu et al. 2019)
***Sporocadus incarnatus***	**CBS 149301**	***Vitis vinifera***	**America**	**OP038025**	**OP076913**	**OP095241**	-	**OP079858**	(Travadon et al. 2022)
***Sporocadus italicus***	**MFLUCC 14-1196**	***Crataegus* sp.**	**Italy**	**MF614831**	**MF614829**	-	**MF943155**	-	(Hyde et al. 2017)
***Sporocadus kurdistanicus***	**CBS 143778**	***Vitis vinifera***	**Iran**	**MW361950**	**MW361958**	-	**MW375356**	**MW375350**	(Moghadam et al. 2022)
*Sporocadus kurdistanicus*	CBS 149023	*Vitis vinifera*	Cyprus	ON695891	ON853905	-	ON863784	ON695852	
*Sporocadus kurdistanicus*	L164	*Vitis vinifera*	Cyprus	ON695892	ON697292	-	ON863786	ON695847	
*Sporocadus kurdistanicus*	CBS 149022	*Vitis vinifera*	Cyprus	ON695890	ON853906	-	ON863785	ON695853	
***Sporocadus lichenicola***	**MFLUCC 14-0052**	***Rosa canina***	**Italy**	**KT005515**	**KT005514**	-	-	-	(Senanayake et al. 2015)
*Sporocadus lichenicola*	CBS 354.90	*Fagus sylvatica*	Germany	MH554035	MH554252	MH554948	MH554470	MH554711	(Liu et al. 2019)
*Sporocadus lichenicola*	NBRC 32625	*Rosa canina*	England	MH883643	MH883646	MH883647	MH883644	MH883645	
***Sporocadus mali***	**CBS 446.70**	***Malus sylvestris***	**Netherlands**	**MH554049**	**MH554261**	**MH554960**	**MH554484**	**MH554725**	(Liu et al. 2019)
*Sporocadus microcyclus*	CBS 887.68	*Ribes* sp.	Netherlands	MH554068	MH554280	MH554981	MH554504	MH554744	
***Sporocadus multiseptatus***	**CBS 143899**	***Viburnum* sp.**	**Serbia**	**MH554141**	**MH554343**	**MH555047**	**MH554576**	**MH554814**	
***Sporocadus pseudocorni***	**MFLU 13-0529**	***Cornus* sp.**	**Italy**	-	**KU359033**	-	-	-	(Li et al. 2016)
*Sporocadus rosarum*	CBS 113832	*Rosa canina*	Sweden	MH553970	MH554189	MH554864	MH554388	MH554629	(Liu et al. 2019)
*Sporocadus rosigena*	CBS 182.50	*Pyrus communis*	Netherlands	MH554013	MH554233	MH554926	MH554447	MH554689	
*Sporocadus rosigena*	L105	*Vitis vinifera*	Cyprus	ON680683	ON692428	-	ON863789	ON695846	(Kanetis et al. 2022)
*Sporocadus rosigena*	CBS 149021	*Vitis vinifera*	Cyprus	ON679667	ON853907	-	ON863787	ON695854	
*Sporocadus rosigena*	CBS 149020	*Vitis vinifera*	Cyprus	ON679668	ON853908	-	ON863788	ON695855	
***Sporocadus rotundatus***	**CBS 616.83**	***Arceuthobium pussilum***	**Canada**	**MH554060**	**MH554273**	**MH554974**	**MH554496**	**MH554737**	(Liu et al. 2019)
***Sporocadus sorbi***	**CBS 160.25**	-	-	**MH554008**	**MH554229**	**MH554924**	**MH554442**	**MH554684**	
*Sporocadus spiniger*	ROC 119	*Rosa omeiensis*	China	OK655791	OK560382	OL742166	OL814548	OM401670	(Peng et al. 2022)
*Sporocadus spiniger*	ROC 120	*Rosa omeiensis*	China	OK655792	OK560383	OL742167	OL814549	OM401671	
*Sporocadus tibetensis*	CGMCC 3.23172	-	China	OR357756	OR247946	OR380979	OR361503	OR381094	(Razaghi et al. 2024)
*Sporocadus tibetensis*	LC15858	-	China	OR357757	OR247947	OR380980	OR361504	OR381095	
***Sporocadus trimorphus***	**CBS 114203**	***Rosa canina***	**Sweden**	**MH553977**	**MH554196**	**MH554876**	**MH554395**	**MH554636**	(Liu et al. 2019)
*Truncatella angustata*	CBS 393.80	*Gevuina avellana*	Chile	MH554041	MH554254	-	MH554476	MH554717	(Cimmino et al. 2021)
*Truncatella angustata*	CJA35	***Vitis vinifera***	Iran	MW361953	-	-	-	-	(Moghadam et al. 2022)
*Truncatella angustata*	CJA82	***Vitis vinifera***	Iran	MW361954	-	-	-	-	

### ﻿Phylogenetic analyses

Phylogenetic trees were constructed using maximum likelihood (ML) and Bayesian inference (BI) methods. Maximum likelihood analysis was performed using RAxML-HPC2 on XSEDE, implemented in the CIPRES web portal (http://www.phylo.org/portal2/) (Stamatakis et al. 2014). The RAxML rapid bootstrapping algorithm was performed using 1,000 pseudoreplicates. The best-fit evolutionary models for individual and combined datasets were estimated under the Akaike Information Criterion (AIC) using jModeltest v. 2.1.10 on the CIPRES web portal for posterior probability (Nylander 2004). The GTR model was the best model for all the datasets. Bayesian inference analyses were conducted by using MrBayes v. 3.2.6 on the CIPRES web portal (Ronquist and Huelsenbeck 2003). Simultaneous Markov chains were run for ten million generations, and trees were sampled every 100^th^ generation. The final phylogenetic trees were visualized in FigTree v. 1.4.4 (Rambaut 2018) and edited in Adobe Illustrator CS v. 6.

### ﻿Pathogenicity assays of fungal isolates

*Sporocadus changchunensis* (EMFCC 0014) and *Sporocadus crataegicola* (EMFCC 0015) were used for the pathogenicity test. Colonised mycelium plugs were obtained from the periphery of fungal colonies cultured on PDA at 25 °C for two weeks under dark conditions (Bhunjun et al. 2021). Healthy leaves of *Crataegus pinnatifida* were surface-sterilized by rinsing with 70% ethanol for 1 min and 0.4% sodium hypochlorite for 30 s (Maniasa et al. 2020). The samples were rinsed three times with sterile distilled water and then allowed to air-dry. The leaves were wounded using sterile forceps for inoculation. Uncolonized plugs were used for the control leaves. All the inoculated samples were incubated individually in petri dishes that contained moistened sterile absorbent cotton wool to create a humid environment, and incubated at room temperature for 21 days. The disease infection index was calculated following Bhunjun et al. (2021). Koch’s postulates were confirmed by morphological characteristics and sequencing. This experiment was repeated in triplicate.

## ﻿Results

### ﻿Phylogenetic analyses

**Figure 1.**
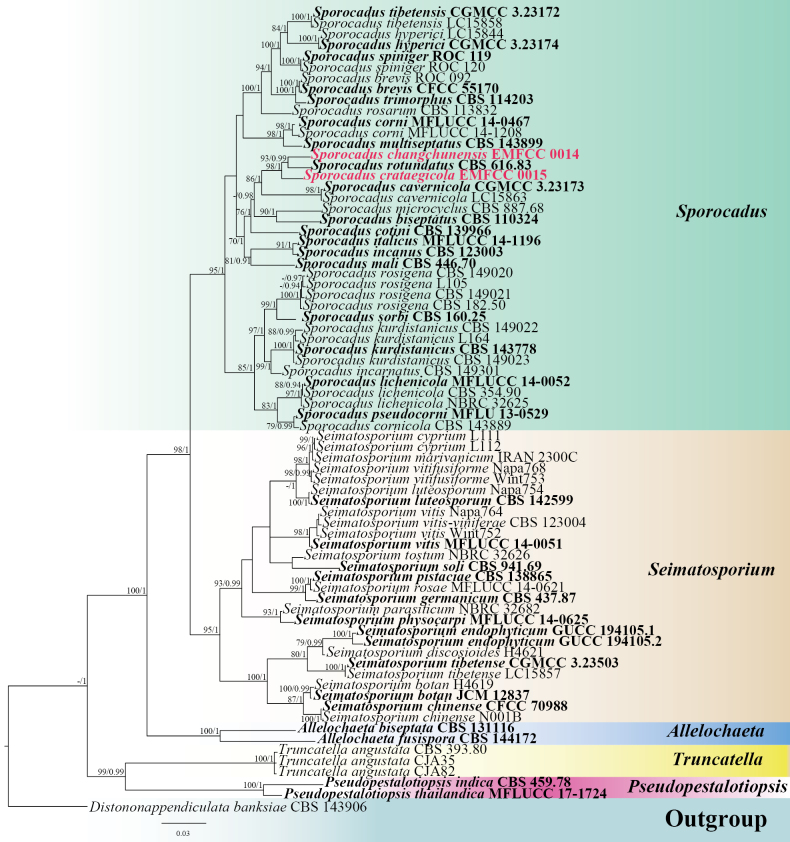
The Bayesian 50% majority-rule consensus phylogram, based on a concatenated ITS, LSU, *rpb*2, *tef*1-α, and *tub*2 dataset of Sporocadaceae. The tree is rooted with *Distononappendiculata banksiae* (CBS 143906). RAxML bootstrap support values ≥70% (ML, left) and Bayesian posterior probabilities ≥0.90 (BPP, right) are shown near the nodes. The new isolates are indicated in red. The type strains are in bold.

The aligned dataset of ITS, LSU, *rpb*2, *tef*1-α, and *tub*2 sequences consisted of 71 strains and 3,684 characteristics, including gaps (ITS: 1–554, LSU: 555–1,420, *rpb*2: 1,421–2,252, *tef*1-α: 2,253–2,865, and *tub*2: 2,866–3,684). The average standard deviation of the split frequencies of the BI analysis was 0.001431, and 5601 trees were sampled after the 20% burn-in. The best-scoring RAxML tree had a final ML optimization likelihood value of -29010.274576. The matrix contained 1,700 distinct alignment patterns, with 28.74% of the characters being undetermined or gaps. Estimated base frequencies were: A = 0.2505, C = 0.2543, G = 0.2444, T = 0.2508; substitution rates AC = 1.4542, AG = 3.7904, AT = 1.2862, CG = 1.1817, CT = 6.3580, GT = 1.0000; proportion of invariable sites I = 0.4505 and gamma distribution shape parameter α = 0.7558. Maximum Likelihood (ML) and Bayesian Inference (BI) analyses produced phylograms with similar topologies (Fig. 1). The BI-derived phylogram was selected to illustrate the phylogenetic relationships among representative Sporocadaceae taxa (Fig. 1). Our two isolates clustered within *Sporocadus*, forming distinct lineages from existing species. *Sporocadus changchunensis* (EMFCC 0014) and *S. rotundatus* (CBS 616.83) formed a closely related clade with strong statistical support (93% ML and 0.99 BPP). *Sporocadus crataegicola* (EMFCC 0015) formed a sister taxon to *S. changchunensis* and *S. rotundatus* with strong statistical support (98% ML and 1.00 BPP).

### ﻿Taxonomy

#### ﻿ Sporocadus changchunensis

Taxon classificationFungiAmphisphaerialesSporocadaceae

R. Xu, W. X. Su, Phukhams. & Y. Li sp. nov.

161496BC-9A66-5CDD-8323-4B7F4C447EB1

859645

Fig. 2

##### Etymology.

Refers to the type location, Changchun City.

**Figure 2.**
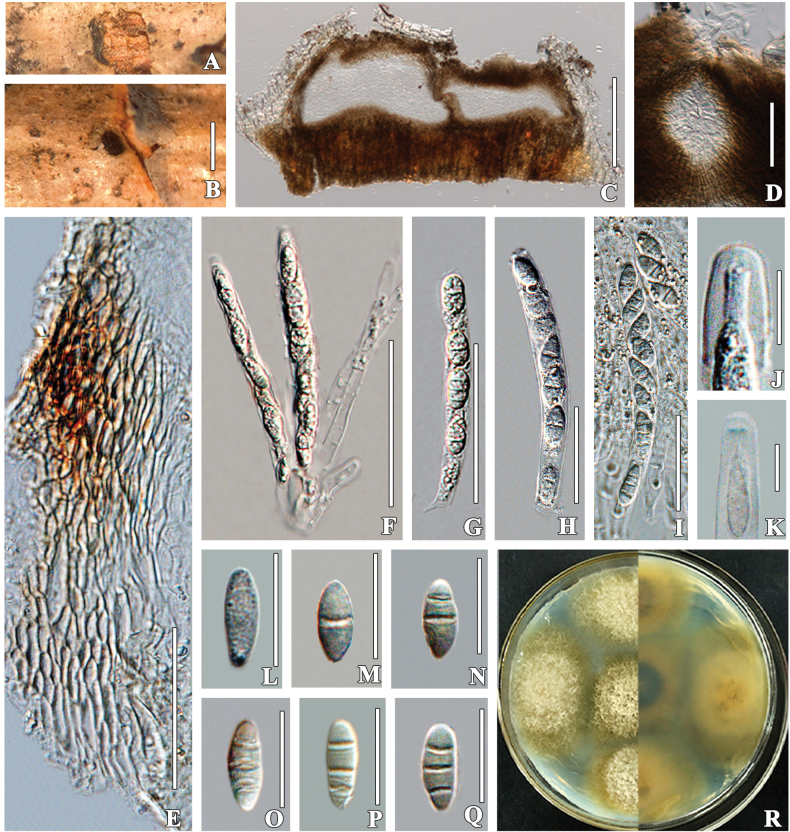
*Sporocadus changchunensis* (HMJAU 60190, holotype). A, B. Ascomata on host surface; C. Vertical section through partial ascoma; D. Ostioles; E. Partial peridium; F. Pseudoparaphyses; G–I. Asci; J. Apical ring (in water); K. Apical ring stained in Melzer’s reagent; L–Q. Development stages of ascospores; R. Culture characteristics on PDA. Scale bars: 500 µm (B); 50 µm (D); 20 µm (E–I); 10 µm (L–Q); 5 µm (J, K).

##### Description.

***Saprobic*** on dried stems of ***Crataegus pinnatifida***. **Sexual morph: *Ascomata*** 184–251 × 279–385 μm (x̄ = 225 × 331 μm, n = 5), single or gregarious, scattered, submerged, depressed globose to globose, visible as light black circles or bark-colored bumps host surface. ***Ostiolar canal*** 72–80 × 44–53 μm (x̄ = 75 × 50 μm, n = 5), cylindrical, sulcate, protruding fillen with periphyses. ***Peridium*** 15–47 μm wide, thin-walled, composed of 4–10 wall layers, outer part comprising brown cells of ***textura angularis***, inner layer thin-walled, brown from the outside radiating to hyaline towards the inside. ***Hamathecium*** of dense, 4–7 μm (x̄ = 5 μm, n = 10) wide, filamentous, septate, rounded at the apex, cellular paraphyses surrounding asci. ***Asci*** 71–88 × 7–8 μm (x̄ = 79 × 7 μm, n = 10), 4–8 ascospores, bitunicate, fissitunicate, broad cylindrical, some curved, short-pedicellate, apically rounded or slightly pointed with J+ ring. ***Ascospores*** 9–16 × 5–8 μm (x̄ = 13 × 6 μm, n = 30), uniseriate, partially overlapping, fusiform to oval, primary septum median, slightly asymmetrical, with 0–3 transverse septa, without vertical septa, hyaline, surface verrucous, without mucilaginous sheath. **Asexual morph**: Undetermined.

##### Culture characteristics.

Colonies on PDA, reaching 2 cm diam after 7 days at 25 °C. Culture from above, dense mycelium, sparse in the periphery, light yellow in the hyphae, light green in the edge of the colony, with three concentric circles on the back of the colony, black or yellow in the center, white to light green radiating outwards, round.

##### Material examined.

China • Jilin Province: Changchun, dead stem of *Crataegus pinnatifida* Bunge, 20 February 2022, W.X. Su and C. Phukhamsakda, HMJAU 60190 (**holotype**); ex-type, EMFCC 0014.

##### GenBank accession numbers.

ITS = OR791294, LSU = OR791292, *rpb*2 = PQ096954, *tef*1-α = PQ096031, *tub*2 = PQ122556.

##### Notes.

In the BLASTn search, the ITS region of strain EMFCC 0014 showed 98.43% similarity to *S. lichenicola* (CBS 446.70) with 99% query cover, translating to 97.45% similarity. Comparatively, the LSU gene region displayed 99.43% similarity to *S. lichenicola* (CBS 160.25) with 99% query cover, translating to 98.44% similarity, while the closest match of the *tub*2 sequence with 94.57% similarity was *S. rotundatus* (CBS 616.83). *Sporocadus changchunensis* (EMFCC 0014) formed a sister clade to *S. rotundatus* (CBS 616.83) with strong bootstrap support (93% ML and 0.99 BPP). The base pair differences between the two species are 3 bp in ITS, 29 bp in *tef*1-α, 20 bp in *rpb*2, and 35 bp in *tub*2. The new isolates also share a close phylogenetic affinity to *S. cavernicola*. However, there is no report for the sexual morph of *S. cavernicola* and *S. rotundatus* (Liu et al. 2019; Razaghi et al. 2024). Morphologically, *Sporocadus changchunensis* differs from *S. italicus*, which was also found on *Crataegus*, by its narrower ostiolar canal (75 × 50 vs. 80 × 75 µm), wider hamathecium (4–7 vs. 2.6–4.1 μm), and smaller asci (71–88 × 7–8 vs. 102–129 × 8.5–10 μm) (Hyde et al. 2017). Additionally, the ascospores of *S. italicus* are ellipsoidal-fusiform or ampulliform, multi-seriate, and sometimes contain one vertical septum, whereas those of *S. changchunensis* are fusiform to oval, uniseriate, and without a vertical septum (Hyde et al. 2017). Hence, we introduce *S. changchunensis* as a novel species, and this is the first report of *Sporocadus* species in Northeast China.

#### ﻿ Sporocadus crataegicola

Taxon classificationFungiAmphisphaerialesSporocadaceae

R. Xu, S. Q. Tian & Phukhams. & Y. Li sp. nov.

B16531C9-BD15-5E50-AD48-AE9FBD4E0E83

859646

Fig. 3

##### Etymology.

Refers to the host genus, *Crataegus*.

**Figure 3.**
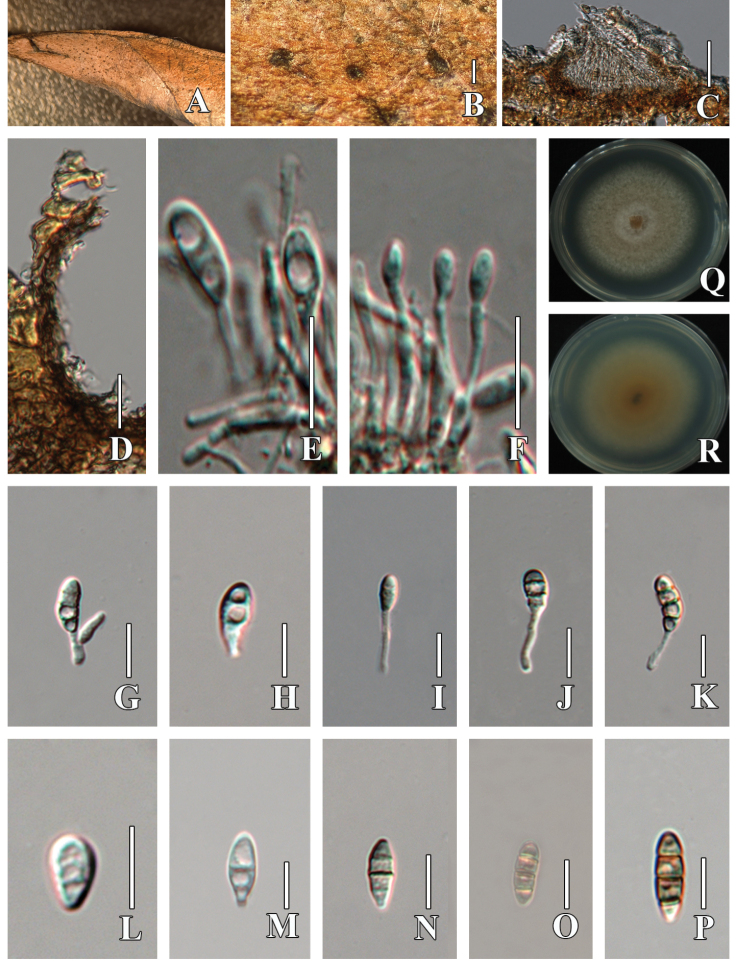
*Sporocadus crataegicola* (HMJAU 60191, holotype). A. Leaf of *Crataegus pinnatifida*; B. Conidiomata on host tissue; C. Section of partial conidioma; D. Close-up of the peridium; E–K. Conidiogenous cells and conidia; L–P. Conidia; Q, R. Culture characteristics on PDA. Scale bars: 100 µm (B, C); 10 µm (D–P).

##### Description.

***Saprobic*** on dead leaves of ***Crataegus pinnatifida***. **Sexual morph**: Undetermined. **Asexual morph: *Conidiomata*** 58–73 × 106–148 (x̄ = 64.8 × 123.7 μm, n = 5), acervular, unilocular, subglobose, superficial to sub-epidermal, solitary, light brown, with a conspicuous apapillate ostiolate. ***Conidiomata wall*** (10–15 µm) composed of brown cells. ***Conidiophores*** 14.8–18.6 µm, long, cylindrical, branched, hyaline, smooth-walled. ***Conidia*** 12–17.5 × 4.5–7 μm (x̄ = 14.71 × 5.6 μm, n = 30), fusiform, straight, infrequently slightly curved, initially hyaline, pale brown at maturity, with 0–3 transverse septa, constricted at the septa, smooth-walled, with thick walls pale brown at maturity, narrowly rounded at both ends, lacking appendages; basal cell obconic with a truncate base, subhyaline to pale brown, 2–4 μm long (x̄ = 3.3 µm), with two median cells subcylindrical to doliform, with thick verruculose walls, pale brown, 6–9 μm long (x̄ = 7.4 µm), with apical cell conical and rounded at apex, pale brown, cylindrical to subcylindrical, 3–5.5 μm long (x̄ = 4 µm).

##### Culture characteristics.

Colonies on PDA, reaching 3–4 cm after 7 days at 25 °C. Culture from above, circular, filamentous, dense, fluffy, rough surface, grayish white, flat with abundant and flocculent aerial hyphae, entire edge; reverse orange at the center, and pale yellow towards the edge.

##### Material examined.

China • Jilin Province: Changchun, dead leaves of *Crataegus pinnatifida* Bunge, 29 June 2022, Rong Xu, S.Q. Tian and C. Phukhamsakda, HMJAU 60191 (**holotype**); ex-type, EMFCC 0015.

##### GenBank accession numbers.

ITS = OR791295, LSU = OR791293, *rpb*2= PQ096955, *tef*1-α= PQ096032.

##### Notes.

A BLASTn search of the ITS region of strain EMFCC 0015 showed a high query cover and similarity (98.79%) to *S. corni* MFLUCC 14-0467. The LSU sequence showed 99.54% similarity to *S. lichenicola* (CBS 160.25) across 98% of the query sequence, which translates to 97.55% similarity. The closest *rpb*2 and *tef*1-α matches were *S. rotundatus* (CBS 616.83) with 95.99% similarity and 95.24% similarity, respectively. The morphology of the conidiomata, conidia, and median cells of our isolate fits well with the descriptions of the asexual morph of *Sporocadus* (Nag Raj 1993; Liu et al. 2019). Phylogenetic analyses of combined ITS, LSU, *rpb*2, *tef*1-α, and *tub*2 datasets (Fig. 1) show that *S. crataegicola* (EMFCC 0015) formed a distinct clade and clustered with *S. changchunensis* and *S. rotundatus* with 98% ML and 1.00 BPP. Conidia of *S. rotundatus* are clavate, obovoid, ellipsoid, or cylindrical (with rounded ends) and have 1–4 septa, whereas those of our isolate are fusiform with 0–3 septa. The asexual morph of *S. changchunensis* remains undetermined. Notably, *Sporocadus crataegicola* differs from *S. changchunensis* in 7/560 bp in ITS, 10/875 bp in LSU, 28/819 bp in *rpb*2, and 27/452 bp in *tef*1-α. Thus, we describe our isolate as a new taxon herein.

### ﻿Pathogenicity test

**Figure 4.**
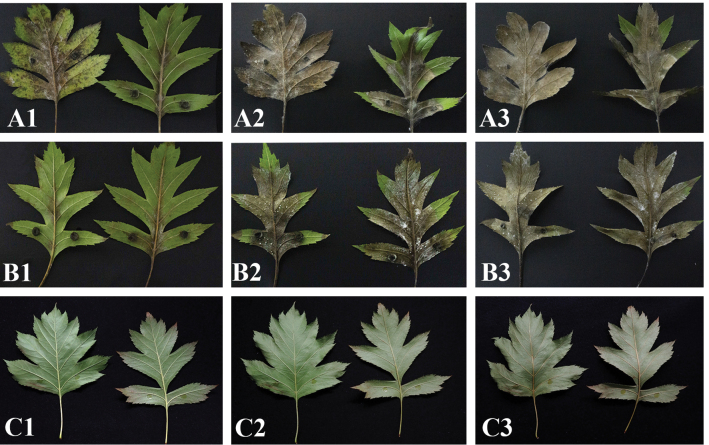
Symptoms of leaves 7, 14, and 21 days after inoculation, respectively. A1–A3. Symptoms of inoculation with *Sporocadus changchunensis* after 7, 14, and 21 days (wounded (left) and non-wounded (right)); B1–B3. Symptoms of inoculation with *Sporocadus crataegicola* after 7, 14, and 21 days (wounded (left) and non-wounded (right)); C1–C3. Control leaves after 7, 14, and 21 days.

The inoculation of *Sporocadus changchunensis* (EMFCC 0014) on the leaves resulted in a yellow-brown color change, characterized by a large number of black spots and a small number of white hyphae in the early stages of the disease, as observed 7 days after inoculation (Fig. 4A1). After 14 days of inoculation, the leaves turned dark brown, covered with white hyphae, and the sporophyte developed (Fig. 4A2). After 21 days of inoculation, the entire leaves were covered with white hyphae, producing a large number of ascomata (Fig. 4A3). In the non-wounded method, only black spots appeared at the inoculation site after 7 days of inoculation (Fig. 4A1). After 14 days of inoculation, the middle of the leaves showed a dark brown colour with a small number of white hyphae, while the leaf margins remained healthy (Fig. 4A2). After 21 days of inoculation, the leaves curled up and were covered with a large number of white hyphae, and only a small portion of the leaf margins was healthy (Fig. 4A3). These findings indicate that *S. changchunensis* has the potential to be pathogenic to hawthorn, as it can infect the host through wounds.

After 7 days of inoculation with *S. crataegicola* (EMFCC 0015), the leaf margins change to light brown (Fig. 4B1). After 14 days of inoculation, most of the leaves were dark brown with a small number of white hyphae and a few conidiomata (Fig. 4B2). After 21 days of inoculation, all the leaves turned dark brown and produced a large number of conidiomata (Fig. 4B3). In the non-wounded method, a small number of white hyphae were observed in the center of the petiole after 7 days of inoculation (Fig. 4B1). After 14 days of inoculation, most of the leaves were brown, with more white hyphae but fewer conidiomata compared to the wounded method (Fig. 4B2). After 21 days of inoculation, all the leaves had turned dark brown, with more white hyphae, whereas the conidiomata were still fewer in number compared to the wounded method (Fig. 4B3). The experiment showed that *S. crataegicola* can infect hawthorn with strong pathogenicity in both wounded and non-wounded methods.

The re-isolated strain was identified as *S. changchunensis* and *S. crataegicola* based on morphological characteristics and sequencing, confirming Koch’s postulates. The disease infection index was 63.13% for *S. changchunensis* on wounded leaves after 7 days, significantly higher than that on non-wounded leaves (11.79%). After 14 days, the disease infection index was 100% on wounded leaves, compared to 43.35% on non-wounded leaves. Almost all the samples showed necrosis after 21 days (Table 2). For *S. crataegicola*, the disease infection index on wounded leaves was 9.77% after 7 days, which was lower than that on non-wounded leaves (16.64%). The disease infection index reached 67.72% on wounded leaves after 14 days, whereas the disease severity was 70.76% on non-wounded leaves. After 21 days, necrosis was observed in almost all samples (Table 2).

**Table 2.** Disease infection index for *Sporocadus changchunensis* and *Sporocadus crataegicola* on hawthorn leaves (wounded (left) and non-wounded (right)).

Species	7d		14d		21d	
	Wounded	Non-wounded	Wounded	Non-wounded	Wounded	Non-wounded
*Sporocadus changchunensis*	63.13%	11.79%	100%	43.35%	100%	93.06%
*Sporocadus crataegicola*	9.77%	16.64%	67.72%	70.76%	100%	98.12%

## ﻿Discussion

In this study, two taxa were isolated from the leaf and woody litter of *C. pinnatifida* in the temperate region of China. Phylogenetic analyses were conducted using concatenated data (ITS, LSU, *rpb*2, *tef*1-α, and *tub*2) for members of the Sporocadaceae. The phylogeny presented here demonstrates that *S. changchunensis* formed a separate clade closely related to *S. rotundatus* (CBS 616.83), a species originally isolated from *Arceuthobium pussilum* in Canada (Liu et al. 2019). The sexual morph of *S. rotundatus* has not been reported, limiting direct morphological comparison. *Sporocadus crataegicola* produces conidia that are 0–3-septate and lack appendages. The conidia of *S. crataegicola* are smaller compared to species without appendages like *S. pseudocorni* (12–17.5 × 4.5–7 vs. 31–42 × 5–7 μm) (Li et al. 2016). Additionally, *S. crataegicola* conidia are pale brown, whereas those of *S. lichenicola* are pale brown to dark brown at maturity (Norphanphoun et al. 2015). Both new species formed an independent phylogenetic branch, distinct from previously described *Sporocadus* species. Consequently, we report two new pestalotioid species of *Sporocadus* from northern China based on multi-locus phylogeny coupled with morphology.

This is the first report of saprophytic and potentially pathogenic *Sporocadus* species isolated from hawthorn in Jilin Province, China. *Sporocadus* is an important genus within Sporocadaceae (Liu et al. 2019), which is commonly associated with various dicotyledon hosts, such as Cornaceae, Fagaceae, Hypericaceae, Rosaceae, Viburnaceae, and Vitaceae (Moghadam et al. 2022; Tanaka et al. 2011; Senanayake et al. 2015; Liu et al. 2019; Peng et al. 2022; Razaghi et al. 2024). Several *Sporocadus* species have been recently found in association with grapes (*Vitis vinifera*), either as part of their mycobiome or as causative agents of grapevine trunk disease (Liu et al. 2019; Raimondo et al. 2019; Kanetis et al. 2022; Moghadam et al. 2022; Travadon et al. 2022; Kolařík et al. 2023). The members of *Sporocadus* are widely distributed across temperate regions in Europe and North America (i.e., America, Canada, Cyprus, England, Germany, Italy, Netherlands, Russia, Serbia, Spain, and Sweden), and China (Serdani et al. 2010; Tanaka et al. 2011; Senanayake et al. 2015; Li et al. 2016; Hyde et al. 2017; Liu et al. 2019; Kanetis et al. 2022; Moghadam et al. 2022; Peng et al. 2022; Razaghi et al. 2024). In this study, our new isolates were collected from Changchun, a city located in temperate regions, consistent with previous studies.

*Crataegus pinnatifida* is a common medicinal and edible plant in the family Rosaceae with broad distribution in China (Zhang et al. 2022; Li et al. 2023b). It can be eaten raw or preserved to make fruit cakes. They are also used in traditional medicine and hold ecological and agricultural importance. However, hawthorn trees are susceptible to fungal pathogen invasion, causing yield and commercial losses. If not controlled in a timely manner, they can spread extensively. Recent fungal investigations have indicated that *Sporocadus italicus* could be a saprobe on the bark of *Crataegus* (Hyde et al. 2017). Saprophytic fungi can transform into pathogens that infect plants and evolve in the complex adaptation process of plant biomass degradation and pathogenesis (Sahu et al. 2023; Bhunjun et al. 2024b). Thus, our investigation into latent pathogens revealed that both new *Sporocadus* species cause leaf blight and etiolation in hawthorn through artificial inoculation. However, this is only the first step in controlling the disease. Further studies should investigate the physiological characteristics of these pathogenic fungi, identify environmental factors that promote disease development, and develop effective disease management strategies for hawthorn cultivation.

## Supplementary Material

XML Treatment for Sporocadus changchunensis

XML Treatment for Sporocadus crataegicola

## ﻿Additional information

### Use of AI

No use of AI was reported.

### Author ORCIDs

Rong Xu https://orcid.org/0000-0002-7744-6321

Wenxin Su https://orcid.org/0000-0002-5470-5853

Shangqing Tian https://orcid.org/0000-0003-4758-3023

Chitrabhanu S. Bhunjun https://orcid.org/0000-0001-8098-3390

Yu Li https://orcid.org/0000-0003-4966-701X

Chayanard Phukhamsakda https://orcid.org/0000-0002-1033-937X

### Data availability

All of the data that support the findings of this study are available in the main text or Supplementary Information.

## Supplementary materials

Supplementary material 1 Phylogram generated from maximum likelihood analysis based on combined ITS, LSU, rpb2, *tef*1-α and tub2 data Data type pdf https://binary.pensoft.net/file/1413727This dataset is made available under the Open Database License (http://opendatacommons.org/licenses/odbl/1.0/). The Open Database License (ODbL) is a license agreement intended to allow users to freely share, modify, and use this Dataset while maintaining this same freedom for others, provided that the original source and author(s) are credited.Rong Xu, Wenxin Su, Shangqing Tian, Chitrabhanu S. Bhunjun, Yu Li, Chayanard Phukhamsakda
