# Supplementary figures and images for: ﻿Morphology and phylogeny reveal two new species of pestalotioid fungi associated with hawthorn in Northeast China
Source: MycoKeys. 2025 Sep 11;122:149–68. doi: 10.3897/mycokeys.122.153767 (PMC12447080; doi:10.3897/mycokeys.122.153767)

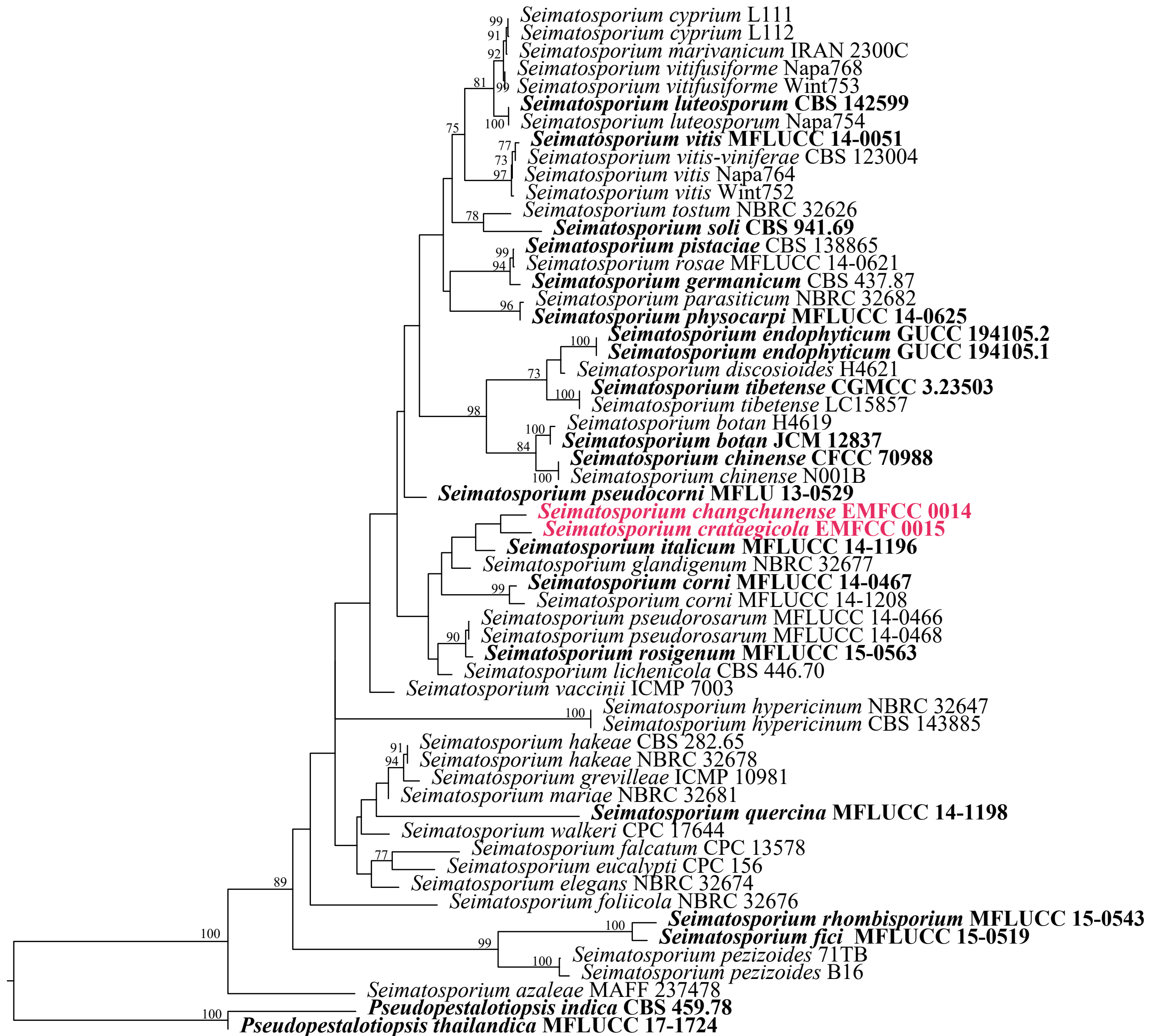

0.06

Supplement: Supplementary material 1 — Phylogram generated from maximum likelihood analysis based on combined ITS, LSU, rpb2, tef1-α and tub2 data [file mycokeys-122-149-s001.pdf]
